# Association between Multidimensional Prognostic Index and Hospitalization and Mortality among Older Adults with Chronic Kidney Disease on Conservative or on Replacement Therapy

**DOI:** 10.3390/jcm9123965

**Published:** 2020-12-07

**Authors:** Silvia Lai, Maria Ida Amabile, Sandro Mazzaferro, Giovanni Imbimbo, Anna Paola Mitterhofer, Alessandro Galani, Filippo Aucella, Giuliano Brunori, Paolo Menè, Alessio Molfino, The Study Group on Geriatric Nephrology of the Italian Society of Nephrology (SIN)

**Affiliations:** 1 Department of Translational and Precision Medicine, Sapienza University of Rome, 00185 Rome, Italy; marida.amabile@gmail.com (M.I.A.); imbimbo.1638090@studenti.uniroma1.it (G.I.); annapaola.mitter@uniroma1.it (A.P.M.); alessio.molfino@uniroma1.it (A.M.); 2 Department of Cardiovascular, Respiratory, Nephrological, Anesthesiologic and Geriatric sciences, Sapienza University of Rome, 00161 Rome, Italy; sandro.mazzaferro@uniroma1.it; 3 Department of Clinical and Experimental Sciences, University of Brescia, 25123 Brescia, Italy; xelainalag@yahoo.it; 4 Nephrology and Dialysis, IRCCS “Casa Sollievo della Sofferenza”, San Giovanni Rotondo, 71013 Foggia, Italy; f.aucella@operapadrepio.it; 5 Division of Nephrology, Hospital “S. Chiara”, APSS, 38122 Trento, Italy; giuliano.brunori@apss.tn.it; 6 Department of Clinical and Molecular Medicine, Sapienza University of Rome, 00189 Rome, Italy; paolo.mene@uniroma1.it

**Keywords:** multidimensional prognostic index, chronic kidney disease, hemodialysis, peritoneal dialysis, hospitalization, mortality

## Abstract

The prevalence of renal disease is constantly increasing in older adults and a prognostic evaluation by a valid tool may play a key role in treatment management. We aimed to assess the association(s) between the multidimensional prognostic index (MPI) and both the hospitalization and mortality among older adults with renal disease. Patients with chronic kidney disease (CKD) (stage 3–5 KDOQI) and on dialysis were considered. Clinical parameters were registered at baseline and after 2 years. In all the patients, the MPI was calculated and divided into grade 0 (low risk), 1 (moderate risk), and 2 (severe risk). Hospitalizations and mortality were recorded during the follow-up and analyzed according to MPI grade. A total of 173 patients, with a median age of 76 years, on conservative (*n* = 105) and replacement therapy (32 patients on hemodialysis, 36 patients on peritoneal dialysis) were enrolled. Of them, 60 patients were in MPI grade 0, 102 in grade 1, and 11 in grade 2. The median duration of all the hospitalizations was 6 days and the number of deaths was 33. MPI significantly correlated with days of hospitalization (*r* = 0.801, *p* < 0.00001) and number of hospitalizations per year (*r* = 0.808, *p* < 0.00001), which was higher in MPI grade 2 compared to grade 1 (*p* < 0.001) and to grade 0 (*p* < 0.001). We found a significant association between MPI grades and mortality (*p* < 0.001). Our results indicate that MPI was associated with outcomes in patients with renal disease, suggesting that a multidimensional evaluation should be implemented in this clinical setting.

## 1. Introduction

The prevalence of older adults affected by chronic kidney disease (CKD) and requiring renal replacement therapy is high worldwide [1,2]. Currently, assessing functional, cognitive, and nutritional status among older CKD patients appears clinically relevant to stratify the risk to develop end-stage renal disease, even more than using the estimated glomerular filtration rate (eGFR) and proteinuria alone [3,4]. The prognostic evaluation of older adults with CKD is essential for physicians to identify the most appropriate clinical decision-making process for the management, treatment, and prevention of complications as well as to have a realistic expectation for patients and their family members. Studies support the idea that multidimensional assessment represents an important aspect in predicting short- and long-term all-cause mortality in older CKD patients [5,6,7]. This is crucial also in considering the high prevalence of frailty in this population negatively impacting on several outcomes [8].

In this light, the multidimensional prognostic index (MPI) has been found to predict mortality in patients with a variety of acute and chronic clinical conditions [4,9]. In particular, in CKD patients, the MPI was shown to be more accurate in predicting mortality when compared to the eGFR alone [7]. Data from hospital-based cohorts also indicate that using the MPI in addition to eGFR ameliorated the prediction of long-term all-cause mortality in CKD older adults [6]. Moreover, the mortality incidence rate (considering all-cause mortality) is significantly raised with the increasing of the MPI grade [6].

The MPI is based on the assessment of nutritional, cognitive, and functional status as well as on medical and social factors [5]; it is calculated from data obtained from a standardized comprehensive geriatric assessment (CGA), including six different domains such as activities of daily living (ADL), instrumental activities of daily living (IADL), short portable mental status questionnaire (SPMSQ), mini nutritional assessment (MNA), Exton-Smith score (ESS), and cumulative index rating scale (CIRS) in addition to information on medication history and cohabitation [5,6]. All these factors might negatively affect patient outcomes including hospitalization and its duration not only in CKD on conservative management but also during replacement therapy.

For this reason, we aimed to assess the association(s) of the MPI over time with the number of hospitalizations, days of hospitalization, and mortality among patients with CKD aged ≥65 years on conservative and replacement therapy, particularly hemodialysis (HD) and peritoneal dialysis (PD).

## 2. Materials and Methods

The study protocol was approved by the Local Clinical Research Ethics Committee (Sapienza University—Azienda Policlinico Umberto I, Rome, Italy—prot. n. 2517/15). The study conforms to the principles outlined in the Declaration of Helsinki and later amendments and we obtained a written informed consent by each patient before the enrollment.

### 2.1. Study Design and Participants

We performed an observational longitudinal study on clinically stable CKD patients consecutively enrolled from March 2015 to July 2017 at the University Hospital “Policlinico Umberto I” of Rome, Sapienza University of Rome, Italy. This study included CKD patients age ≥65 years on conservative therapy (eGFR ≤ 60 mL/min, stage 3–5 KDOQI), or replacement therapy (HD or PD) for at least 3 months. Statins, antihypertensive and antiplatelet therapies, and/or therapies with calcium, calcitriol and phosphate binders were continued in all patients included in the study. We recorded the clinical history and excluded patients with acute cerebrovascular and cardiovascular events within 3 months before the study, history of malignancy, or degenerative neurological or psychiatric diseases. We did not enroll patients who were not able to sign the informed consent or refused to give consent, nor did we enroll patients with missing data to calculate the MPI. We also excluded patients who were planning at the enrollment to relocate to another nephrology unit/dialysis center within the next 6 months.

The eGFR was calculated with abbreviated modification of diet in renal disease formula [10]. Clinical and laboratory variables, including hemoglobin, serum vitamin D, intact parathyroid hormone (iPTH) albumin, electrolytes, pH and base excess, were recorded at baseline and at 12 and 24 months in all CKD patients, and among HD patients, they were registered during the middle of the week, whereas among PD patients, before the first replacement of the morning with an empty peritoneum at routine visit [11]. Finally, body weight was determined to the nearest 0.1 kg using a calibrated digital scale. Body mass index (BMI) was calculated using the formula of (weight (kg)/height^2^ (m^2^)).

### 2.2. Calculation of the MPI at Baseline

We calculated the MPI as established in previous studies, consisting as a product of the CGA [5], which included data from 6 standardized scales: ADL and IADL, exploring the functional status; SPMSQ, exploring the cognitive status; MNA, investigating nutritional status; ESS for mobility and risk of pressure sore; CIRS, for multi-morbidity assessment; in addition, the number of drugs to assess polypharmacy, and co-habitation status were recorded for a total of 63 items [4,9]. The MPI was calculated in all the participants from the integrated total scores and expressed as 3 risk classes: grade 0 = low risk (MPI value between 0 and 0.33), grade 1 = moderate risk (MPI value between 0.34 and 0.66) and grade 2 = severe risk (MPI value ranging from 0.67 to 1.00) [7,9].

### 2.3. Hospitalization and Mortality over the 24-Month Follow-Up

All the participants were followed up with for 24 consecutive months after the enrollment and over this period we recorded the total number of days of hospitalizations per year and the number of annual admissions as well as the number of deaths per number of patients [12].

### 2.4. Statistical Analysis

Data management and analysis were performed using IBM^®^ SPSS^®^ Statistics 20.0 for Windows^®^ software (IBM Corporation, New Orchard Road Armonk, New York, NY, USA). The normality of variables was tested using the Shapiro–Wilk method for normal distributions. All continuous variables were expressed as mean ± standard deviation, categorical variables were expressed as number (percentage). The comparison of the data of patients, for all quantitative variables considered, was performed using non-parametric Wilcoxon test and Student’s *t* test. For comparing proportions was applied chi-square test. Student’s t-test or the Mann–Whitney U-test were performed to determine differences between groups. The binomial test or chi-square test was used for comparison of categorical data. Pearson’s correlation was used to determine, in bivariate correlation, the relationship and the strength of association between the variables, considering all the patients together and also based on the stage of the disease for hospitalization and mortality (CKD 3, CKD 4-5 or replacement therapy, including HD and PD patients). A value of *p* < 0.05 was considered statistically significant.

## 3. Results

### 3.1. Patients’ Characteristics at Baseline

We initially considered 177 patients; 2 patients refused to give consent and 2 patients were excluded because they transferred to other nephrology units during the study period, making complete data unavailable. Therefore, a total of 173 patients (107 male), with a median age of 76 (70; 80) years were consecutively included; they were affected by CKD on conservative therapy (stage 3–5 KDOQI) (*n* = 105, 72 male), on HD (*n* = 32 patients, 15 male) and on PD (*n* = 36 patients, 20 male). The patients’ characteristics are shown in Table 1.

### 3.2. MPI Classes

The MPI score was calculated at baseline and all the participants were divided into 3 risk classes: grade 0 (low risk) = 60 patients (35%), grade 1 (moderate risk) = 102 patients (59%), and grade 2 (severe risk) = 11 patients (6%).

CKD patients on conservative therapy among moderate and severe MPI risk classes (1 and 2) were 72/105 (69%), HD patients were 10/32 (31%) and patients in PD were 31/36 (86%).

### 3.3. 24-Month Follow-Up and Clinical Characteristics

Among the entire cohort, we registered an average 206 hospitalization per year.

At the end of the follow-up, 33 patients died (*n* = 21 of CKD, *n* = 4 of HD, *n* = 8 of PD). Therefore, a total of 140 patients with a median age of 73 years (68.75; 79) were studied at 24 months and their characteristics are shown in Table 1.

### 3.4. MPI and Hospitalization

**Figure 1:**
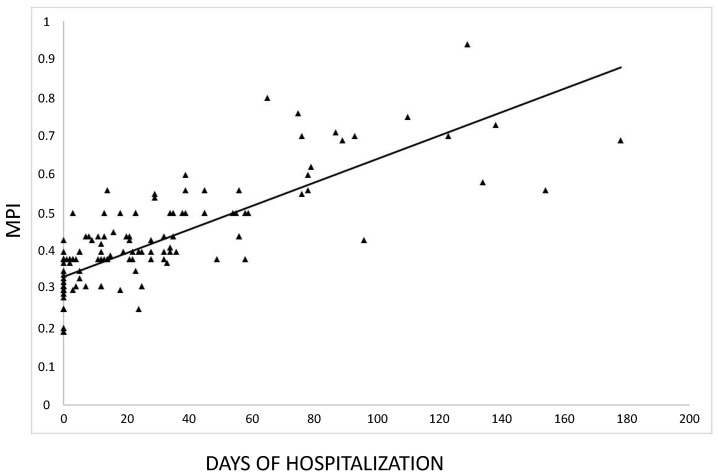
Correlation between the multidimensional prognostic index (MPI) score and days of hospitalization over the 24-month follow-up. (*r* = 0.801, *p* <0.00001).

We found a significant positive correlation between MPI and total number of days of hospitalization registered over the 24-month follow-up (*r* = 0.801, *p* < 0.00001) (Figure 1) as well as between MPI and the number of hospitalizations per year (*r* = 0.808, *p* < 0.00001).

**Figure 2:**
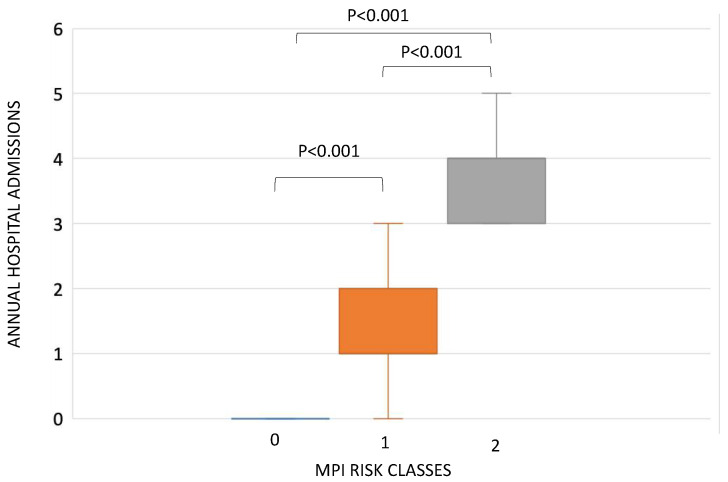
Differences in annual hospital admission between multidimensional prognostic index (MPI) risk class 0 (0, IQR 0; 0), MPI risk class 1 (1, IQR 1; 2) and MPI risk class 2 (4 IQR 3; 4).

According to MPI risk classes, the mean of annual hospital admissions was different between the three MPI grades (*p* < 0.001) (Figure 2). In particular, patients in MPI risk class 2 showed a significantly higher median number of hospitalizations per year (4, IQR 3; 4) with respect to patients in MPI risk class 1 (1, IQR 1; 2) (*p* < 0.0001) and to those in MPI risk class 0 (0, IQR 0; 0) (*p* < 0.0001) as well as a higher median number of hospitalizations per year between patients in MPI risk class 1 versus those with risk 0 (*p* < 0.0001) (Figure 2). These significant differences between MPI risk classes were confirmed when considering separately CKD patients in non-replacement therapy (*p* < 0.0001), and when considering CKD patients in replacement therapy only (*p* < 0.0001).

Moreover, all the six domains comprised in the MPI significantly correlated with the total number of days of hospitalization and with the numbers of annual hospital admissions over the 24-month follow-up (Table 2).

### 3.5. MPI and Mortality

We found a significant association between MPI and the number of deaths for all risk classes (χ^2^ = 61.22, *p* < 0.0001), and the analysis of standardized residuals (positive or negative) showed that the differences were statistically significant in each MPI risk class (*r* < 1.96 or *r* > 1.96) (Table 3).

Moreover, a significant association between MPI and the number of deaths for all risk classes was also documented when considering separately CKD patients in non-replacement therapy (*p* < 0.0001) and CKD patients in replacement therapy (HD + PD) (*p* = 0.002).

## 4. Discussion

The prognostic evaluation of older adults with CKD is crucial in the decision analysis of care processes to evaluate the most appropriate management and treatment of patients with renal disease. In particular, older adults often present several comorbidities, and life expectancy is likely to be influenced by a multitude of factors. The prognosis of older adults with CKD is strongly affected by functional, cognitive, and nutritional status, psychosocial capacity, treatments, and other factors that are directly or indirectly related to the disease, suggesting the need of a prognostic tool which should be accurate in predicting mortality risk, with the objective of developing an overall comprehensive plan for treatment and follow-up [5,13]. This may be also important to decide if replacement therapy should be initiated or not. Based on this concept, the MPI was successfully used in CKD to predict all-cause mortality. Interestingly, in our study, we observed a significant association between MPI classes and outcomes not only in patients with CKD on conservative therapy (stage 3–5 KDOQI) but also in patients on HD and PD. In fact, patients on replacement therapy are known to be frail with important clinical implications [14]. In particular, among our cohort, the 86% of patients on PD were those in MPI risk classes 1 and 2.

MPI significantly correlated with the number of days of hospitalization during the 2-year follow-up, and this observation was also confirmed when analyzing the correlation between MPI and the number of hospitalizations per year. Based on these results, we believe that physicians should pay particular attention to patients within MPI risk class 2 considering that these patients were those with the highest number of hospitalizations per year. Interestingly, when considering each of the six domains comprised in the MPI, all of them significantly correlated with the total number of days of hospitalization and with hospitalizations per year. In particular, a high correlation was documented for the ADL and hospitalizations. This observation appears novel in the literature considering that the majority of the data available regarding ADL and outcomes, specifically on HD or PD, were focused on mortality [15] and not on hospitalization rate. Frailty assessment was also shown to be useful for decision-making among CKD patients on conservative care [16].

Moreover, ADL is a sensitive instrument in predicting frailty in the renal population, especially in patients in HD, and among frail patients a significantly higher rate of hospitalization was observed [17].

In this light, our data are clinically important and potentially useful for physicians in assessing the length of stay and the potential increased healthcare-related costs of patients with CKD and on replacement therapy. During our follow-up, we confirmed the significant association between MPI and mortality (number of deaths) for all the risk classes. Interestingly, all the patients identified at baseline in MPI class 2 died during the 24-month follow-up. This highlights the clinical relevance of the MPI in predicting mortality among patients with worse nutritional, cognitive, and functional status as well as with negative medical and social factors. In this view, our data are in accordance with the ones reported by others, where MPI risk classes were significantly associated with mortality in patients with renal disease [5,18]. In particular, our results may add novel information on MPI and outcomes, considering that they were obtained in a cohort of renal patients that included CKD patients at different stages and patients on dialysis, although not equally distributed in each group, followed in the same nephrology unit. This tool may be clinically useful to identify accurate and more adequate management of patients with renal disease.

Our study focused on MPI and outcomes also in patients on replacement therapy, considering the clinical relevance of predicting hospitalizations and survival in HD and PD patients. In this light, recent data showed that HD patients with frailty incurred higher healthcare costs with respect to those without frailty over a mean follow-up of 2.3 years [8]. In addition, we have previously shown that HD patients may present neurological and psychological dysfunctions that were associated with impaired quality of life [19]. Moreover, cognitive impairment was highly associated with a frail phenotype [20], resulting in increased costs and early mortality [21].

MPI calculation may also be important in PD patients to predict outcomes considering that, in this setting, we have previously found some alterations of nutritional and metabolic status, specifically among older adults (aged ≥65 years) [22].

Noteworthy is the fact that MPI includes, among others, the assessment of co-habitation status, CIRS, and polypharmacy. In particular, the use of several medications represents a clinical issue highly associated with poor prognosis during the course of several chronic diseases in the elderly [23].

In the systematic assessment of prognostic indices for all-cause mortality in older patients, the MPI has been proved to be the only selected mortality index based on a multidimensional approach, indicating the clinically relevant impact of the multidimensional derangement on the risk of mortality [5]. Interestingly, an approach that includes the assessment of several factors [5] appears challenging and may potentially add important information for clinical care in the elderly population affected by renal disease.

Our study has several limitations. We have included a highly heterogenous population consisting of patients with both moderate CKD and end-stage renal disease. The number of the participants in each group (in particular, in CKD patients in the stage 4–5 group and in the replacement group) was small, and for this reason, we could not also analyze the associations among HD and PD separately. We acknowledge that the epidemiology of the patients with CKD at earlier stage and of patients with more advanced renal disease (CKD stage 5 and dialysis) is different, likely limiting the interpretation of our results. Patients were also recruited from a single center. The implementation of MPI requires time to complete the collection of information to calculate the score and, therefore, it may limit its feasibility in clinical practice. More importantly, MPI is a tool using several items of self-reported information that may not represent an objective assessment.

## 5. Conclusions

In the present study, MPI was significantly associated with hospitalization and mortality over 24-month follow-up in older adults with CKD on conservative treatment or renal replacement therapy. This suggests that MPI may be clinically useful to assess prognosis in this setting and that physicians should pay attention to a multidimensional evaluation aimed at reducing patients’ morbidity and mortality.

## Figures and Tables

**Table 1:** Patient characteristics. Total patients at baseline were *n* = 173 and at month 24 were *n* = 140.

	Baseline	Month 24
Male, *n* (%)	107 (62)	84 (60)
Age, y	76 (70; 80)	73 (69; 79)
BMI, kg/m^2^	26.4 ± 4.4	23.9 ± 3.4
Hemoglobin, g/dL	11.6 (10.6; 13)	12.6 (11.6; 13.4)
Serum creatinine, mg/dL		
All	2.1(1.8; 6.0)	2.8 (1.9; 7.2)
CKD stage 3 ^*^	1.8 (1.4; 2)	2.08 (1.49; 2.5)
CKD stage 4–5 ^#^	1.9 (1.8; 2.2)	2.63 (2.03; 2.9)
Replacement therapy ^§^	6.3 (5.6; 7)	9 (7.53; 11)
Total serum nitrogen, mg/dL		
All	97 (69; 138)	99 (87; 131)
CKD stage 3 ^*^	73 (64; 95)	89 (67; 102)
CKD stage 4–5 ^#^	76.5 (58; 101)	98 (76; 115)
Replacement therapy ^§^	147 (114; 179)	122 (99; 148)
eGFR, mL/min/1.73 m^2^		
All	27.5 (10; 39)	21 (8.2; 30.3)
CKD stage 3 ^*^	39 (35; 52)	30 (25; 39)
CKD stage 4–5 ^#^	25 (22; 28)	25 (19.8; 29.3)
Replacement therapy ^§^	8.7 (7.3; 10.7)	7.1 (5.5; 8.8)
pH	7.33 (7.30; 7.38)	7.37 (7.33; 7.40)
Base excess	−2.50 (−6.00; 1.00)	−1.90 (−3.50; −0.80)
Sodium, mEq/L	139 (137; 142)	140 (139; 143)
Potassium, mEq/L	4.79 ± 0.63	4.63 ± 0.62
Albumin, mg/dL		
All	3.98 (3.5; 4.1)	4.60 (4.2; 5.0)
CKD stage 3 ^*^	3.89 (3.45; 4.1)	4.67 (4.4; 5.0)
CKD stage 4–5 ^#^	3.8 (3.5; 4)	4.7 (4.5; 5.1)
Replacement therapy ^§^	4.0 (3.8; 4.4)	3.8 (3.4; 4.2)
iPTH, pg/mL	160.5 (58.5; 165)	109.5 (76.0; 221.3)
25-OH-VitD, ng/mL	21.5 (14.4; 29.3)	14.8 (7; 20)
SBP, mmHg	130 (120; 140)	130 (120; 140)
DBP, mmHg	80 (70; 86)	80 (70; 85)

Abbreviations: BMI, body mass index; CKD, chronic kidney disease; eGFR, estimated glomerular filtration rate; iPTH, intact parathyroid hormone; SBP, systolic blood pressure; DBP, diastolic blood pressure. Median (25th; 75th) is shown for non-normally distributed variables. * at baseline *n* = 77 and at month 24 *n* = 63; ^#^ at baseline *n* = 28 and at month 24 *n* = 21; ^§^ at baseline *n*= 68 and at month 24 *n* = 56.

**Table 2:** Correlations between each of the six domains of the multidimensional prognostic index (MPI) and the days of hospitalization and the number of hospitalizations per year.

	Days of Hospitalization	*n*° of Hospitalizations per Year
Each of the 6 domains of MPI		
ADL		
*r*	−0.629	−0.573
*p*-value	<0.00001	<0.00001
IADL		
*r*	−0.544	−0.572
*p*-value	<0.00001	<0.00001
SPMSQ		
*r*	−0.419	−0.381
*p*-value	<0.00001	<0.00001
EXTON-SMITH		
*r*	−0.476	−0.480
*p*-value	<0.00001	<0.00001
CIRS		
*r*	0.19	0.232
*p*-value	0.013	0.002
MNA		
*r*	−0.533	−0.585
*p*-value	<0.00001	<0.00001

Abbreviations: ADL, activities of daily living; IADL, instrumental activities of daily living; SPMSQ, short portable mental status questionnaire; CIRS, cumulative index rating scale; MNA, mini nutritional assessment.

**Table 3:** Association between mortality (death no/yes) and multidimensional prognostic index (MPI) by risk classes. (Risk class 0 = MPI between 0 and 0.33; Risk class 1 = MPI between 0.34 and 0.66; Risk class 2 = MPI between 0.67 and 1.00).

			MPI Risk Class			Total (*n*)
			0	1	2	
**Death**	NO	Count	60	80	0	140
		Expected count	48.6	82.5	8.9	140.0
		Standardized residual	4.7	−1.0	−7.1	
	YES	Count	0	22	11	33
		Expected count	11.4	19.5	2.1	33.0
		Standardized residual	−4.7	1	7.1	
**Total**		Count	60	102	11	173
Person’s Chi-square			Value 61.22		*p*-value < 0.0001	
